# The Potential Role of Regulatory Genes (*DNMT3A*, *HDAC5*, and *HDAC9*) in Antipsychotic Treatment Response in South African Schizophrenia Patients

**DOI:** 10.3389/fgene.2019.00641

**Published:** 2019-07-10

**Authors:** Kevin Sean O’Connell, Nathaniel Wade McGregor, Robin Emsley, Soraya Seedat, Louise Warnich

**Affiliations:** ^1^Department of Genetics, Stellenbosch University, Stellenbosch, South Africa; ^2^Department of Psychiatry, Stellenbosch University, Tygerberg, South Africa

**Keywords:** schizophrenia, epigenetics, neuropsychiatric genetics, gene expression, treatment response

## Abstract

Despite advances in pharmacogenetics, the majority of heritability for treatment response cannot be explained by common variation, suggesting that factors such as epigenetics may play a key role. Regulatory genes, such as those involved in DNA methylation and transcriptional repression, are therefore excellent candidates for investigating antipsychotic treatment response. This study explored the differential expression of regulatory genes between patients with schizophrenia (chronic and antipsychotic-naïve first-episode patients) and healthy controls in order to identify candidate genes for association with antipsychotic treatment response. Seven candidate differentially expressed genes were identified, and four variants within these genes were found to be significantly associated with treatment response (*DNMT3A* rs2304429, *HDAC5* rs11079983, and *HDAC9* rs1178119 and rs11764843). Further analyses revealed that two of these variants (rs2304429 and rs11079983) are predicted to alter the expression of specific genes (*DNMT3A*, *ASB16*, and *ASB16-AS1*) in brain regions previously implicated in schizophrenia and treatment response. These results may aid in the development of biomarkers for antipsychotic treatment response, as well as novel drug targets.

## Introduction

The onset of schizophrenia is marked by a first psychotic episode, typically followed by subsequent relapse episodes, separated by intervals of remission (Lieberman et al., 2001). Diagnosis remains difficult due to a heterogeneity of symptoms as well as symptom overlap with other disorders (Tandon et al., 2009). Furthermore, treatment strategies are not optimal (Brandl et al., 2014), and it is estimated that approximately half of all patients with schizophrenia will not respond satisfactorily to antipsychotics (Lohoff and Ferraro, 2010), which are the mainstay of treatment and, as such, widely used. They are effective for positive symptoms (such as delusions and hallucinations); however, their efficacy for negative symptoms (such as apathy, anhedonia, and social withdrawal) is limited (Leucht and Davis, 2017). Moreover, antipsychotics are known to result in a number of adverse drug reactions (ADRs), including motor abnormalities (Chowdhury et al., 2011) and metabolic deficits (Tandon et al., 2010). These ADRs are often severe and long lasting, resulting in reduced compliance and diminished positive outcomes (Brandl et al., 2014). Considering the high rate of non-responders to treatment and the potential severe side effects of treatment, there is a clear need to improve our understanding of antipsychotic treatment response.

Pharmacogenetics, the study of the effects of genetic variation on treatment outcomes, has been moderately successful in explaining variability in inter-individual treatment response. Variation within the dopaminergic pathway has been extensively investigated, and several variants within dopamine receptor genes are associated with treatment response (Lencz et al., 2010) and ADRs (Bakker et al., 2008). In addition, variation within genes encoding drug-metabolizing enzymes has yielded similar findings of association (Lohoff and Ferraro, 2010; Brandl et al., 2014). Larger hypothesis-free-driven genome-wide association studies (GWAS) have also associated common variation with antipsychotic treatment response (Liou et al., 2012; Zhang and Malhotra, 2013); however, there has been little validation or replication of these associations, and their biological relevance remains to be determined (Liou et al., 2012; Zhang and Malhotra, 2013). In addition to these challenges, the majority of treatment response heritability is not explained by common variation, suggesting that other factors must also play a role (Manolio et al., 2009). This underscores the complexity and multi-factorial nature of treatment response since common and rare genetic factors, environmental factors, and gene-environment interactions need to be considered (Manolio et al., 2009; Majchrzak-Celińska and Baer-Dubowska, 2017).

Epigenetics refers to molecular mechanisms that determine inherited cellular phenotypes without alteration of the genotype (Wu et al., 2012). These mechanisms include various molecular processes, such as histone modification, nucleosome remodeling, non-coding RNAs, and DNA methylation (Wu et al., 2012). Both unique and overlapping altered epigenetic modifications are associated with schizophrenia etiology and pathogenesis as well as antipsychotic treatment (Swathy and Banerjee, 2017). As such, an extremely complex, multi-directional relationship needs to be considered in studying the role of epigenetics in treatment response (pharmacoepigenetics), since genes that are implicated may be regulated by epigenetic modification independent of disease etiology, treatment outcomes, and/or ADRs (Kurita et al., 2012; Melas et al., 2012; Tang et al., 2014; Majchrzak-Celińska and Baer-Dubowska, 2017). For example, alterations of DNA methylation profiles (Melas et al., 2012; Tang et al., 2014) and altered chromatin structure (Kurita et al., 2012) may influence treatment response.

Although specific gene–environment interactions are required for epigenetic modifications to occur, it is important to note that specific genes directly or indirectly produce proteins and other products necessary for these modifications. For example, the *DNMT1* gene encodes for Dnmt1, which is responsible for the maintenance of existing methylation patterns during cell division (Bostick et al., 2007), while *DNMT3A* and *DNMT3B* encode for the enzymes responsible for establishing *de novo* methylation patterns (Okano et al., 1999). Regulatory genes, such as those involved in DNA methylation and transcriptional repression, are therefore excellent candidates for investigation with regards to antipsychotic treatment response.

The aims of this study were, therefore, to identify candidate regulatory genes that may be involved in antipsychotic treatment response, and then to determine if variation within these genes is associated with treatment outcome. In order to address these aims, we first sought to identify regulatory genes that were differentially expressed between patients with schizophrenia (including chronically medicated patients and drug-naïve first-episode patients) and healthy controls. By identifying genes differentially expressed between the chronically medicated patients and both the drug-naïve first-episode patients and healthy controls, we identified a subset of candidate genes that may be implicated in treatment response independent of schizophrenia pathogenesis. Variation within these candidate genes was then investigated in an independent cohort for association with treatment response over time.

## Materials and Methods

**Figure 1:**
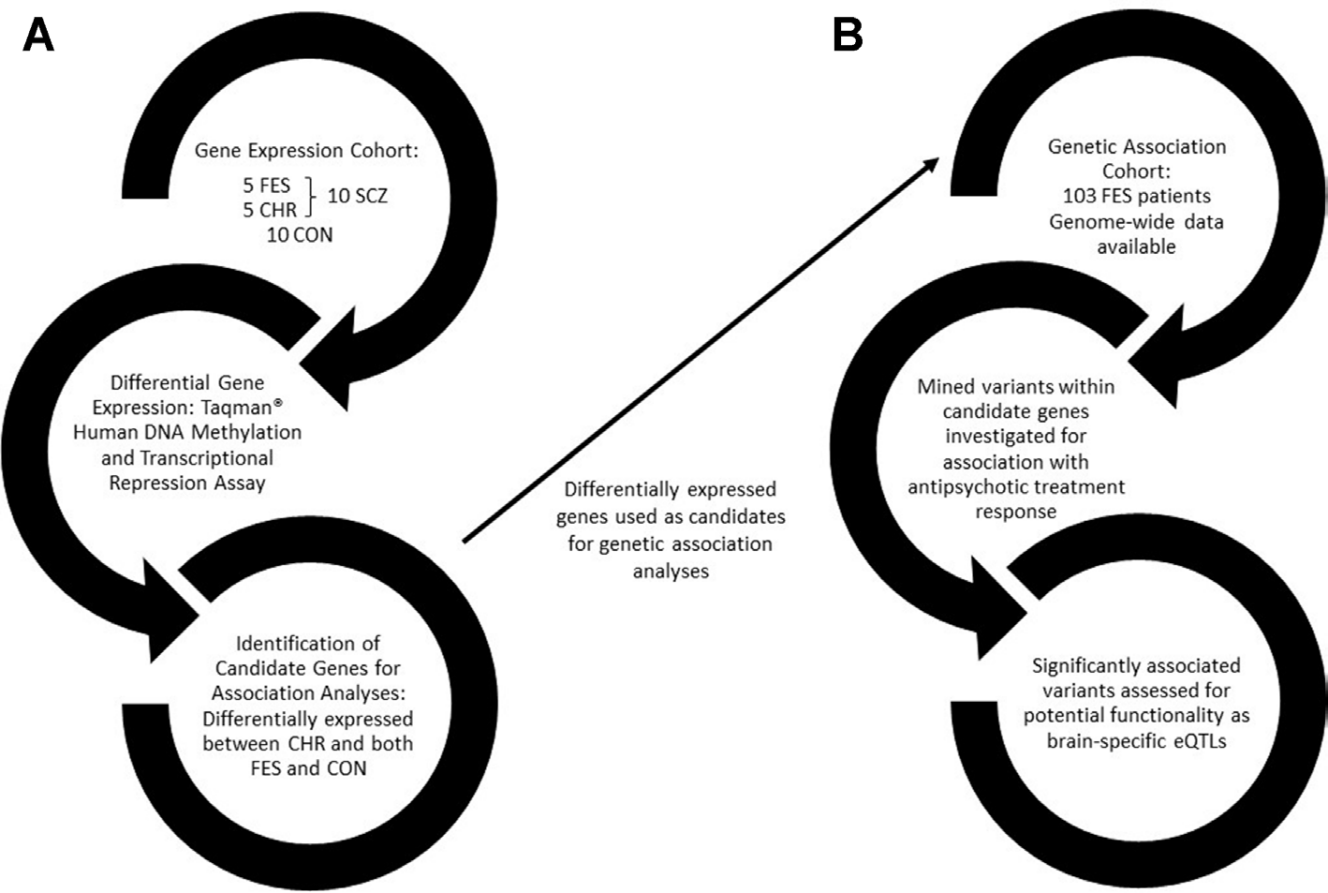
A flow diagram outlining the approach used in this study. **(A)** The first arm of the study involves identifying differentially expressed genes between healthy controls and schizophrenia patients, further divided into drug-naïve first-episode patients and chronic patients. **(B)** Differentially expressed genes were then used as candidates for genetic association with antipsychotic treatment response in a drug-naïve FES cohort. FES, first episode schizophrenia; CHR, chronic schizophrenia; SCZ, schizophrenia; CON, healthy control; eQTLs, expression quantitative trait loci.

An outline of the approach used to perform this study is provided in **Figure 1**.

### Participants for Gene Expression Analyses

The cohort consisted of 20 unrelated, age-matched, and male South African participants of South African colored (SAC) descent [10 patients with schizophrenia (SCZ), 26.4 ± 7.9 years old, and 10 healthy controls (CON), 26.5 ± 7.6 years old]. Patients with SCZ were further divided into two equal groups consisting of five first-episode patients (FES) (24.8 ± 10.9 years old) and five chronic patients (CHR) (28.0 ± 4.1 years old). The FES patients were recruited and sampled within 1 week of their first episode of psychosis and were subsequently administered flupenthixol decanoate (Fluanxol, Lundbeck, Copenhagen, Denmark). The CHR patients were recruited 6.2 ± 0.4 years after their first episode, and all were treated with flupenthixol decanoate (Fluanxol, Lundbeck, Copenhagen, Denmark) in addition to other psychotropic medications (**Supplementary Table 1**). All patients were diagnosed using the *Diagnostic and Statistical Manual of Mental Diseases*, Fourth Edition, Text Revision (DSM-IV TR) (American Psychiatric Association, 2000) diagnostic criteria for SCZ, schizophreniform disorder, or schizo-affective disorder. Written informed consent was obtained from all patients, or their caregivers, prior to the study, and ethical approval was granted by the Human Research and Ethics Committee (HREC), Faculty of Medicine and Health Sciences, Stellenbosch University (N13/08/115).

### Total RNA Isolation and cDNA Synthesis

Whole blood was collected from all participants by venipuncture of a forearm vein into PAXgene Blood RNA tubes (Qiagen, California, USA), which were stored at −20°C until processed. Total RNA was extracted using the PAXgene Blood RNA Kit IVD according to the manufacturer’s instructions (Qiagen, California, USA). All samples were eluted in 80 µl of elution buffer and stored at −80°C until further analysis. RNA yield and quality were assessed using an Agilent Model 2100 Bioanalyzer (Agilent Technologies, California, USA) and a DropSense 16 spectrophotometer (TRINEAN, Belgium). All samples had 260/280 > 2.0 and RIN > 7.0.

Reverse transcription was performed using the High-Capacity cDNA Reverse Transcription Kit with RNase Inhibitor (Applied Biosystems, California, USA), according to the manufacturer’s specifications. Briefly, for each sample, 100 ng of RNA was added to 2 μl of random and oligo (dT) primers in a final reaction of 20 μl. These reaction tubes were then placed in the GeneAmp^®^ PCR Systems 2700 (Applied Biosystems, California, USA) thermocycler at 25°C for 10 min, 37°C for 120 min, and 85°C for 5 min to inactivate the reverse transcriptase enzyme. The cDNA samples were then stored at −20°C until analyzed.

### Quantitative Real-Time PCR

Quantitative real-time PCR (qRT-PCR), to determine the relative mRNA abundance, was performed using the StepOnePlus Real-Time PCR System and SDS Software version 2.3 (Applied Biosystems, California, USA). The commercially available fluorescence-based TaqMan^®^ Human DNA Methylation and Transcriptional Repression microarray plates (Applied Biosystems, California, USA) were used to assess the relative mRNA content of 27 regulatory genes (**Table 1**). Each sample was analyzed in two independent experiments to control for experimental bias using the following PCR conditions: a 10-min heat activation step (95°C) followed by 40 cycles of 15 s at 94°C and 1 min at 63°C. Fluorescence data, indicative of the amount of PCR product, was captured at each cycle. The relative mRNA concentrations were then calculated based on the cycle number that the threshold quantity of PCR product is obtained (Ct). The RefFinder online tool was used to assess the stability of potential housekeeping genes (https://www.heartcure.com.au/reffinder) (Xie et al., 2012), and *GAPDH* was identified as the most stable. Ct values were therefore normalized to values of *GAPDH* and expressed relative to this control (Livak and Schmittgen, 2001).

**Table 1:** Relative expression levels for DNA methylation and transcriptional repression genes between control participants and schizophrenia patients.

Gene	CON	SCZ	p-value^1^	FDR^1^	FES	CHR	p-value^2^	FDR^2^	p-value^3^	p-value^4^	p-value^5^
*B2M* *^#^*	1.053 ± 0.364	1.115 ± 0.471	0.675	0.104	0.852 ± 0.396	1.411 ± 0.373	0.015	**0.002**	0.212	**0.034**	**0.009**
*CH4*	1.045 ± 0.316	1.457 ± 0.438	0.011	**0.004**	1.326 ± 0.368	1.604 ± 0.487	0.006	**0.001**	0.056	**0.003**	0.200
*DNMT1*	1.044 ± 0.314	1.421 ± 0.456	0.027	**0.008**	1.336 ± 0.429	1.517 ± 0.494	0.024	**0.003**	0.063	**0.009**	0.430
*DNMT3A* *^#^*	1.061 ± 0.380	1.392 ± 0.357	0.015	**0.005**	1.208 ± 0.210	1.599 ± 0.384	0.004	**0.001**	0.299	**0.004**	**0.018**
*DNMT3B*	1.064 ± 0.383	1.907 ± 0.870	0.001	**0.001**	1.621 ± 0.626	2.229 ± 1.029	0.001	**0.001**	**0.011**	**0.001**	0.156
*HDAC1* *^#^*	1.046 ± 0.338	1.239 ± 0.374	0.131	0.025	1.063 ± 0.205	1.436 ± 0.433	0.029	**0.004**	0.899	**0.024**	0.035
*HDAC2*	1.087 ± 0.452	1.339 ± 0.463	0.109	0.023	1.346 ± 0.471	1.331 ± 0.486	0.217	0.019	n.d.	n.d.	n.d.
*HDAC3*	1.037 ± 0.306	1.341 ± 0.359	0.016	**0.005**	1.173 ± 0.219	1.531 ± 0.402	0.034	**0.004**	0.084	**0.026**	0.264
*HDAC4*	1.067 ± 0.490	1.154 ± 0.301	0.124	0.025	1.037 ± 0.303	1.284 ± 0.256	0.312	0.026	n.d.	n.d.	n.d.
*HDAC5* *^#^*	1.045 ± 0.329	1.482 ± 0.369	0.012	**0.004**	1.370 ± 0.413	1.626 ± 0.265	0.004	**0.001**	0.165	**0.006**	**0.035**
*HDAC6*	1.053 ± 0.354	1.601 ± 0.557	0.109	0.023	1.401 ± 0.369	1.825 ± 0.666	0.379	0.030	n.d.	n.d.	n.d.
*HDAC7*	1.049 ± 0.330	1.427 ± 0.395	0.001	**0.001**	1.211 ± 0.324	1.669 ± 0.330	0.002	**0.001**	**0.041**	**0.002**	0.211
*HDAC8*	1.056 ± 0.371	1.309 ± 0.362	0.002	**0.001**	1.162 ± 0.221	1.498 ± 0.434	0.002	**0.001**	**0.029**	**0.001**	0.120
*HDAC9* *^#^*	1.049 ± 0.339	1.451 ± 0.441	0.006	**0.003**	1.274 ± 0.503	1.650 ± 0.263	<0.001	** <0.001**	0.250	** <0.001**	**0.011**
*HDAC10*	1.101 ± 0.590	1.274 ± 0.406	0.060	0.015	1.106 ± 0.331	1.463 ± 0.418	0.033	**0.004**	0.441	**0.021**	0.174
*HDAC11*	0.949 ± 0.354	1.286 ± 0.418	0.006	**0.003**	1.176 ± 0.335	1.409 ± 0.488	0.004	**0.001**	0.193	** <0.001**	0.078
*HMBS*	1.078 ± 0.459	1.346 ± 0.397	0.059	0.015	1.185 ± 0.427	1.526 ± 0.287	0.059	**0.006**	n.d.	n.d.	n.d.
*MBD2*	1.041 ± 0.316	1.199 ± 0.381	0.207	0.038	1.029 ± 0.334	1.390 ± 0.353	0.044	**0.005**	0.928	**0.023**	**0.047**
*MBD3* *^#^*	1.041 ± 0.314	1.618 ± 0.598	0.002	**0.001**	1.280 ± 0.300	1.998 ± 0.634	<0.001	** <0.001**	0.077	** <0.001**	**0.008**
*MECP2*	1.070 ± 0.438	1.233 ± 0.472	0.305	0.051	1.037 ± 0.387	1.452 ± 0.482	0.099	**0.009**	n.d.	n.d.	n.d.
*RBBP4*	1.052 ± 0.388	1.162 ± 0.299	0.109	0.023	1.086 ± 0.265	1.246 ± 0.329	0.430	0.033	n.d.	n.d.	n.d.
*RBBP7*	1.051 ± 0.356	1.717 ± 0.547	<0.001	**<0.001**	1.500 ± 0.438	1.962 ± 0.579	<0.001	** <0.001**	**0.010**	** <0.001**	0.082
*RPLP0* *^#^*	1.064 ± 0.412	1.198 ± 0.635	0.480	0.077	0.857 ± 0.531	1.582 ± 0.531	0.011	**0.002**	0.288	**0.015**	**0.013**
*SAP18*	1.047 ± 0.347	1.192 ± 0.379	0.242	0.042	1.056 ± 0.277	1.346 ± 0.436	0.137	0.012	n.d.	n.d.	n.d.
*SAP30*	0.931 ± 0.404	1.507 ± 0.685	0.012	**0.004**	1.644 ± 0.789	1.371 ± 0.591	0.030	**0.004**	**0.034**	0.126	0.535
*SIN3A*	1.057 ± 0.373	1.570 ± 0.404	0.001	**0.001**	1.482 ± 0.381	1.669 ± 0.430	0.002	**0.001**	**0.012**	**0.002**	0.357
*TRDMT1*	1.080 ± 0.428	1.127 ± 0.357	0.807	0.120	1.052 ± 0.330	1.225 ± 0.393	0.651	0.048	n.d.	n.d.	n.d.

Bold typeset indicates significant differences [false discovery rate (FDR) < 0.01 or p < 0.05]. Data are presented as mean ± standard deviation.

CON, control participants; SCZ, schizophrenia patients; FES, first episode schizophrenia patients; CHR, chronic schizophrenia patients; n.d., not determined.

^1^CON vs. SCZ, ^2^CON vs. FES vs. CHR, ^3^CON vs. FES, ^4^CON vs. CHR, ^5^FES vs. CHR. ^#^Genes included for association analyses with treatment response.

### Gene Expression Analyses

Differential gene expression between the CON and SCZ groups was determined using unpaired t-tests or Mann-Whitney U tests where appropriate. Additionally, one-way analysis of variance (ANOVA) and Tukey’s *post hoc* tests were used to determine differences between the CON, FES, and CHR groups. The false discovery rate (FDR) correction (Benjamini and Hochberg, 1995) was used to correct for multiple testing in the CON *vs*. SCZ and CON *vs*. FES *vs*. CHR analyses (27 genes; FDR < 0.01). A significance threshold of p < 0.05 was used for the Tukey’s *post hoc* tests since these were only performed in the case of a significant (FDR < 0.01) ANOVA result. Candidate genes for genetic association analyses with treatment response were selected if they met the following three criteria: i) significant differences in gene expression between the CHR and CON groups, ii) no significant differences in gene expression between the FES and CON groups, and iii) significant differences in gene expression between the CHR and FES groups.

### Participants for Genetic Association Analyses

The patient cohort included 103 unrelated South African FES patients meeting DSM-IV TR (American Psychiatric Association, 2000) diagnostic criteria for SCZ, schizophreniform disorder, or schizo-affective disorder (80% SAC, 12% Xhosa, and 8% European descent) and have been described previously (Drogemöller et al., 2014; Chiliza et al., 2015; Ovenden et al., 2017; O’Connell et al., 2018). All patients received treatment with flupenthixol decanoate (Fluanxol, Lundbeck, Copenhagen, Denmark), a long-acting injectable antipsychotic, according to a fixed protocol. Treatment response was assessed using the Positive and Negative Syndrome Scale (PANSS) (Kay et al., 1987) over a period of 12 months, with measurements taken biweekly for the first 6 weeks, and every 3 months thereafter. Written informed consent was obtained from all patients, or their caregivers, prior to the study, and ethical approval was granted by HREC, Faculty of Medicine and Health Sciences, Stellenbosch University (N06/08/148).

### Genetic Association Analyses

Variants within these candidate genes, obtained from each National Center for Biotechnology Information gene page (https://www.ncbi.nlm.nih.gov/gene/), were mined from available genome-wide genotype data. All 103 FES patients described above were previously genotyped with the Infinium OmniExpressExome-8 Kit (Illumina, California, USA) in accordance with the standard Illumina protocol. Variants were excluded from downstream analysis if they exhibited high rates of missing genotype data (> 5%), if their minor allele frequency (MAF) was < 1% or if they showed departure from the Hardy Weinberg equilibrium (p < 1×10^-4^). For this study, only variants with a minor allele frequency greater than 5% and a call rate greater than 98% were included for the association analyses. Furthermore, only one representative variant was included when two or more variants were shown to be in linkage disequilibrium (LD, r^2^ > 0.6) with one another. In total, 274 variants were included for analyses. All processing of genetic data was performed using Plink v1.9 (Purcell et al., 2007; Purcell and Chang).

Association analyses were conducted in R (R Core Team, 2017) using R packages lme4 (Bates et al., 2014) and lmerTest (Kuznetsova et al., 2016). Linear mixed-effects models were used to investigate the effect of genetic variants on change in PANSS scores for each subscale (positive, negative, and general) and total over the 12-month period, adjusting for age, gender, proportion ancestry, and baseline PANSS scores. Multiple modes of inheritance were investigated and the Bonferroni correction method was used to correct for multiple testing (274 variants, four modes of inheritance, four PANSS domains; threshold p = 1.14 × 10^-5^).

### Bioinformatic Analyses

Significantly associated variants were identified for intronic variants only, which were assessed for potential functionality as brain-specific eQTLs by interrogating the BRAINEAC (www.braineac.org) (Ramasamy et al., 2014) and GTex (www.gtexportal.org) (GTEx Consortium, 2013, GTEx Consortium, 2015) online databases.

## Results

### Relative Gene Expression Results

After the initial analysis, comparing gene expression between the CON and SCZ groups, 14 genes were shown to have significantly different expression between these groups. Specifically, the *CH4*, *DNMT1*, *DNMT3A*, *DNMT3B*, *HDAC3*, *HDAC5*, *HDAC6*, *HDAC7*, *HDAC9*, *HDAC11*, *MBD3*, *RBBP7*, *SAP30*, and *SIN3A* genes showed increased expression in the SCZ group when compared to the CON group (**Table 1**). When comparing the CON group to the FES and CHR groups, 19 genes were shown to be differentially expressed (**Table 1**). *Post hoc* analyses identified seven genes that were significantly over-expressed in the CHR group when compared to both the CON and FES groups.

**Figure 2:**
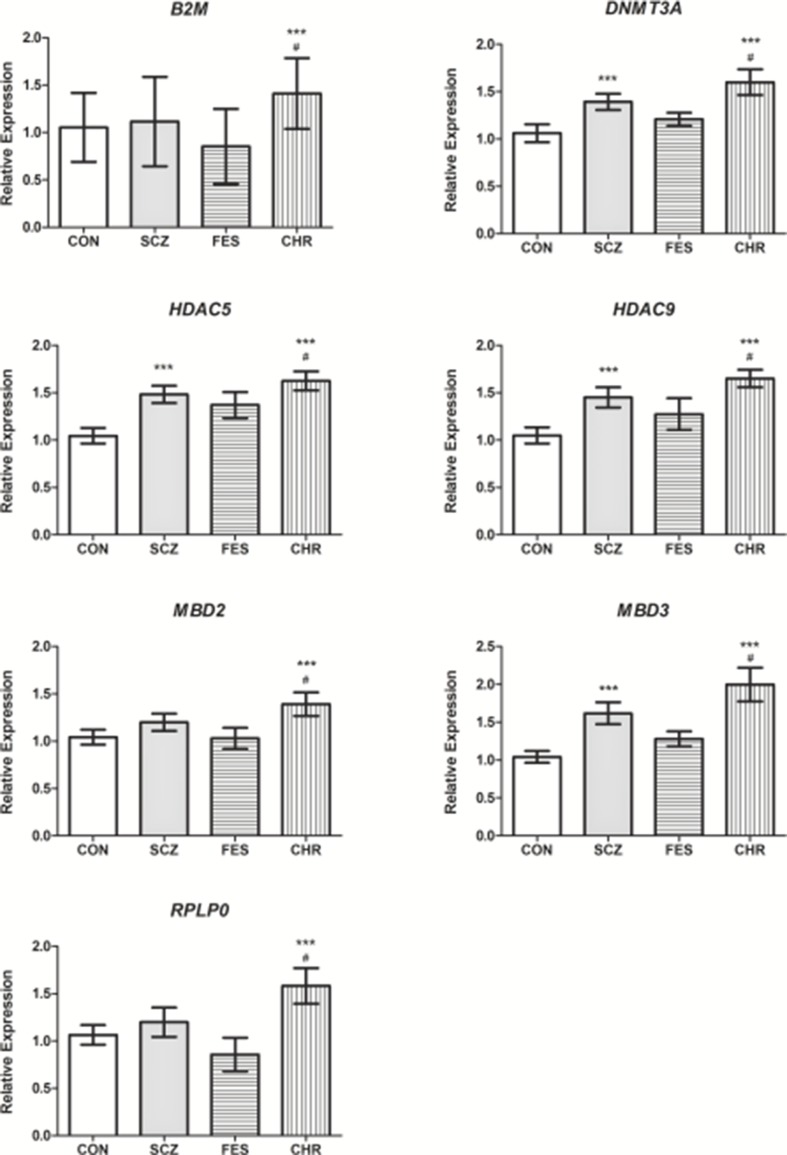
The relative expression levels of *B2M*, *DNMT3A*, *HDAC5*, *HDAC9*, *MBD2*, *MBD3*, and *RPLP0* in the SCZ, FES, and CHR groups when compared to the CON group. ***Significant after correction, *versus* CON group. ^#^Significant after correction, *versus* FES group. Data is presented as mean ± standard error. Additional details available in **Table 1**.

For follow-up investigation as candidates for association with antipsychotic treatment response, seven genes were selected. Specifically, the *B2M*, *DNMT3A*, *HDAC5*, *HDAC9*, *MBD2*, *MBD3*, and *RPLP0* genes were selected since the expression levels of these genes were significantly different between the CHR and CON groups and CHR and FES groups, respectively (**Table 1**, **Figure 2**). Furthermore, no significant differences in gene expression were identified for these genes when comparing the FES and CON groups (**Table 1**, **Figure 2**).

### Genetic Association Results

Of the 274 variants investigated within the seven candidate genes, four were significantly associated with treatment response, as described by a change in PANSS scores over time, when considering correction for multiple testing (p < 1 × 10^-5^) (**Table 2**). All significant associations were identified with the PANSS-negative domain. Specifically, the *DNMT3A* rs2304429 *CC* genotype was significantly associated with an improved treatment response (greater reduction in PANSS-negative scores per month) when compared to the *TC* genotype. The rate of PANSS-N score reduction was significantly faster over time (an additional 1.92% per month) in patients with the *DNMT3A* rs2304429 *CC* genotype when compared to patients with the *TC* genotype. The *HDAC5* rs11079983 *TT* genotype was significantly associated with a poorer treatment response (less reduction in PANSS-negative scores per month) when compared to the *CC* genotype. Patients with the *HDAC5* rs11079983 *TT* genotype had significantly slower rate of PANSS-N score reduction over time (less by 2.36% per month) when compared to patients with the *CC* genotype. Two variants within *HDAC9* (rs1178119 and rs11764843) were also significantly associated with poorer treatment response (less improved PANSS-negative treatment trajectory scores) as shown in **Table 2**. For *HDAC9* rs1178119, patients with the *GA* genotype had a significantly slower rate of reduction in PANSS-N scores over time (less by 2.04% per month) than patients with the *AA* genotype. Presence of the *HDAC9* rs1178119 *G* allele was also significantly associated with a slower rate of reduction in PANSS-N scores over time (less by 1.52% per month per *G* allele). Similarly, a significantly slower rate of reduction in PANSS-N scores over time (less by 1.92% per month) was also identified for patients with the *HDAC9* rs11764843 *CA* genotype when compared to those with the *AA* genotype.

**Table 2:** Variants significantly associated with treatment trajectory for the PANSS negative domain.

Gene	Variant	Mode	Contrast	Effect (% change per month)	95% Confidence Interval	p-value
*DNMT3A*	rs2304429	Genotypic	TC *vs* CC	1.92	1.12 to 2.68	2.50 x10^-6^
*HDAC5*	rs11079983	Genotypic	TT *vs* CC	2.36	1.32 to 3.36	8.41 x10^-6^
*HDAC9*	rs1178119	Genotypic	GA *vs* AA	2.04	1.24 to 2.8	5.42 x10^-7^
		Additive	Each G	1.52	0.88 to 2.16	5.21 x10^-6^
	rs11764843	Genotypic	CA *vs* AA	1.92	1.16 to 2.68	1.22 x10^-6^

### Bioinformatics

The variants significantly associated with change in PANSS scores were assessed using the BRAINEAC (Ramasamy et al., 2014) and GTex (GTEx Consortium, 2013, GTEx Consortium, 2015) databases. Two variants (rs2304429 and rs11079983) were identified as potential brain-specific eQTLs. The rs2304429 variant was suggested by BRAINEAC to alter the expression of *DNMT3A* in particular brain regions—namely, the putamen (PUTM), the cerebellar cortex (CRBL), the temporal cortex (TCTX), and the medulla (MEDU). Specifically, the TT genotype was associated with reduced expression of *DNMT3A* in these brain regions. In addition, the HDAC5 rs11079983 variant was shown to alter the expression of *ASB16* and *ASB16-AS1* in the cerebellum when considering the GTex database. Specifically, the TT genotype is associated with reduced expression of *ASB16* and increased expression of *ASB16-AS1* when compared to the CC genotype, respectively. None of the other variants were identified as brain-specific eQTLs in the BRAINEAC or GTex databases.

## Discussion

We identified a number of DNA methylation and transcriptional repression genes to be significantly over-expressed in patients with SCZ, in first-episode and CHR, when compared to healthy controls (**Table 1**). Specifically, seven candidate regulatory genes were identified, including *B2M*, *DNMT3A*, *HDAC5*, *HDAC9*, *MBD2,* *MBD3*, and *RPLP0*, and variation within these genes was assessed for association with antipsychotic treatment response. Four variants were found to be significantly associated with poorer treatment trajectory in the PANSS-negative domain.

In this study, significant increases in the expression of *DNMT1* and *DNMT3A* were identified between all patients with SCZ and controls; however, further analyses revealed that this increase was only present when considering the CHR patients and not in the FES patients. Increased *DNMT1* and *DNMT3A* gene expression in the GABAergic neurons of SCZ patients has been previously identified (Zhubi et al., 2009), while the increased expression of *DNMT1* was also established in the peripheral blood lymphocytes of patients with SCZ (Auta et al., 2013). When considering these previous studies, it is interesting to note that the mean ages of their study cohorts were 57 ± 11 and 56 ± 18 (Zhubi et al., 2009) and 43.6 ± 10.3 (Auta et al., 2013), respectively. Given the young age of onset of SCZ, it is likely that the patients in these cohorts are more indicative of chronic SCZ and that the results of this study therefore replicate these previous findings.

The expression of a number of *HDAC* genes (1–4, 6, and 9) was previously investigated in the prefrontal cortex of patients with SCZ (Sharma et al., 2008). Only *HDAC1* was shown to have increased expression in patients with SCZ when compared to controls, while no significant differences in expression were identified for the other *HDAC* genes (Sharma et al., 2008). One possible explanation for the differences between these results and those of our study is that these patients were subject to a range of different medications, including typical and atypical antipsychotics, mood stabilizers (including valproic acid), antidepressants, stimulants, and sedatives which all may have an effect on the gene expression observed (Majchrzak-Celińska and Baer-Dubowska, 2017). Of the other differentially expressed genes in this study, *SAP30* expression was previously investigated and no significant changes were identified (Vawter et al., 2006). These results highlight the need for well-defined and deep phenotyping when investigating the molecular etiologies of neuropsychiatric disorders since their molecular architecture is malleable to disorder progression as well as treatment and other environmental factors (Gurwitz and Pirmohamed, 2010).

Due to the nature of the genes investigated in this study, changes in gene expression that were identified are indicative of altered regulatory mechanisms in chronically medicated patients with SCZ (**Table 1**). The alteration of these regulatory mechanisms is likely the result of a combination of disease progression, antipsychotic medication (Swathy and Banerjee, 2017), and the influence of other environmental factors through the process of epigenetics (Feil and Fraga, 2012). To further elucidate these complex interactions, gene expression differences between first-episode and CHR, as well as healthy controls, were assessed. Significantly increased expression of seven genes (*B2M*, *DNMT3A*, *HDAC5*, *HDAC9*, *MBD2*, *MBD3*, and *RPLP0*) was observed in patients with chronic SCZ, when compared to FES and healthy controls (**Figure 2**). These seven genes were selected as candidates for association with antipsychotic treatment response.

Novel associations were identified between variants within *DNMT3A* (rs2304429), *HDAC5* (rs11079983), and *HDAC9* (rs1178119, rs11764843) and antipsychotic treatment response as defined by a change in PANSS scores over time (**Table 2**). Specifically, all four of these variants were associated with a significantly worse treatment trajectory in the negative PANSS symptom domain. Bioinformatics analyses of these variants revealed that two variants are predicted to exert functional changes as eQTLs. The *DNMT3A* rs2304429 and *HDAC5* rs11079983 variants were predicted to alter expression of specific genes in particular brain regions. Specifically, individuals with the rs2304429 *CC* genotype have increased *DNMT3A* gene expression in the PUTM, CRBL, TCTX, and MEDU when compared to individuals with the *TC* or *TT* genotypes. In addition, individuals with the *HDAC5* rs11079983 *TT* genotype have reduced expression of *ASB16* and increased expression of *ASB16-AS1* in the cerebellum when compared to the *CC* genotype. In this study, the rs2304429 *CC* and rs11079983 *CC* genotypes infer improved treatment response indicating that the brain region-specific gene expression changes associated with these variants may play a role. From these results, it may be hypothesized that increased expression of *DNMT3A*, as result of the rs2304429 *CC* genotype, in the abovementioned brain regions may result in *de novo* methylation patterns (Okano et al., 1999) that result in increased efficacy of antipsychotic medication. Similarly, reduced expression of *ASB16* and *ASB16-AS1* in the cerebellum, in the presence of the rs11079983 *CC* genotype, may result in altered ubiquitin-mediated pathways (Kohroki et al., 2005) and cytokine signaling (Babon et al., 2009) with beneficial effects when considering antipsychotic response. These brain regions have previously been implicated in SCZ (Hokama et al., 1995; Turetsky et al., 1995; Harrison, 2004; Yeganeh-Doost et al., 2011; Williams et al., 2014; Tohid et al., 2015) and suggested as targets for treatment (Buchsbaum et al., 2003; Hugdahl et al., 2009; Mitelman et al., 2009; Parker et al., 2014). These brain region-specific eQTLs should therefore be investigated as biomarkers and potential targets for antipsychotic treatment response.

The potential underlying mechanisms of action associated with the remaining two significant findings are not clear. Further studies investigating the exact role that these variants may have in antipsychotic treatment response is warranted. Furthermore, the novel associations identified in this study should be replicated and validated in additional independent cohorts. Moreover, the eQTL data presented were not generated from Sub-Saharan African-ancestry-related individuals and further studies are required to confirm whether the variants presented here have similar eQTL effects in populations of African-ancestry. Due to the small sample sizes used for the gene expression analyses in this study, these results should be interpreted with caution and require replication. Functional studies incorporating *in situ* and *in vivo* assays should also be considered for validation of these results. Moreover, the gene expression results presented in this study should be replicated in female patients with SCZ and controls.

In conclusion, this study identified significant differential expression of DNA methylation and transcriptional repression genes between FES with SCZ, chronically medicated patients with SCZ and healthy controls. Variants within specific differentially expressed genes were significantly associated with antipsychotic treatment response, and highlighted particular brain regions in which altered expression of specific genes may play a role in treatment outcome. These results may aid in the development of biomarkers for antipsychotic treatment response, as well as, novel drug targets, and treatment strategies.

## Ethics Statement

Written and informed consent was obtained from all patients, or their caregivers, prior to the study and ethical approval was granted by the Human Research and Ethics Committee (HREC), Faculty of Medicine and Health Sciences, Stellenbosch University (N13/08/115 and N06/08/148).

## Author Contributions

KO’C NM, and LW conceived the study. KO’C performed the laboratory work and statistical analyses. RE and SS were involved in participant recruitment. KO’C wrote the initial draft for the manuscript. NM, LW, RE, and SS provided detailed critique of the manuscript. All authors approved the final manuscript for submission.

## Funding

This work was supported by the South African Medical Research Council “SHARED ROOTS” Flagship Project Grant no. MRC-RFA-IFSP-01-2013/SHARED ROOTS (Prof Soraya Seedat, Department of Psychiatry, Stellenbosch University) and the National Research Foundation (NRF): KO was funded by the Scarce Skills Post-Doctoral Fellowship (grant no. 96833). LW was funded by the Competitive Program for Rated Researchers (grant no. 93498) and the Bioinformatics and Functional Genomics Program (grant no. 93681). The opinions expressed and conclusions arrived at are those of the authors and are not necessarily attributed to these funding sources.

## Conflict of Interest Statement

The authors declare that the research was conducted in the absence of any commercial or financial relationships that could be construed as a potential conflict of interest.
