# Supplementary material for: The Potential Role of Regulatory Genes (DNMT3A, HDAC5, and HDAC9) in Antipsychotic Treatment Response in South African Schizophrenia Patients
Source: Front Genet. 2019 Jul 10;10:641. doi: 10.3389/fgene.2019.00641 (PMC6635553; doi:10.3389/fgene.2019.00641)
Supplement: Supplementary file 1 [file Table_1.docx]

**Supplementary Table 1.** The antipsychotic and other psychotropic medications administered to the chronic schizophrenia patients (CHR).

| **Participant** | **Medications** |
| --- | --- |
| 1 | Flupenthixol decanoate  Clozapine  Amisulpride  Sodium Valproate |
| 2 | Flupenthixol decanoate  Risperidone |
| 3 | Flupenthixol decanoate  Risperidone  Lorazepam  Promethazine |
| 4 | Flupenthixol decanoate  Citalopram |
| 5 | Flupenthixol decanoate  Clozapine |
